# Two-dimensional graphene nanomaterials for combined photothermal and chemotherapy-enhanced targeted therapy of breast cancer

**DOI:** 10.1016/j.mtbio.2025.101668

**Published:** 2025-03-13

**Authors:** Xiongjie Zhu, Juanjuan Lei, Chao Jiang, Zhaobi Fang, Wenkai Zhang, Zhe Yang, Rui Guo, Rui Xu, Xiaoshan Hu

**Affiliations:** a Department of Medical Oncology, Guangzhou Institute of Cancer Research, The Affiliated Cancer Hospital, Guangzhou Medical University, Guangzhou, China; b Department of Hepatobiliary Surgery, Guangzhou Institute of Cancer Research, The Affiliated Cancer Hospital, Guangzhou Medical University, Guangzhou, China; c Department of Cancer Center, The People's Hospital of Baoan Shenzhen, Guangdong, China; d Department of Oncology, Zhujiang Hospital of Southern Medical University, Guangzhou, China; e Southern Medical University, Guangzhou, China; f Key Laboratory of Biomaterials Of Guangdong Higher Education Institutes, Guangdong Provincial Engineering and Technological Research Centre for Drug Carrier Development, Department of Biomedical Engineering, Jinan University, Guangzhou, China

**Keywords:** Two-dimensional, Photothermal therapy, Chemotherapy, Targeted, Breast cancer

## Abstract

Breast cancer is one of the most common malignant tumors in women, accounting for 7–10 % of all malignant tumors in the body. Second only to uterine cancer, it poses a significant threat to women's health. In this study, a two-dimensional material based on GO nanoparticles was designed to combine photothermal therapy (PTT) with chemotherapy for targeted treatment of breast cancer. Firstly, HPAA was grafted onto GO through an amide reaction after EDC/NHS activation. Secondly, RGD was attached to the amino group of HPAA. Finally, the chemotherapeutic drug doxorubicin was loaded through intermolecular interactions. *In vitro* results showed that the HPAA/GO-RGD@DOX exhibited a good photothermal effect and drug release profile, with greater drug release in acidic environments compared to neutral ones. Additionally, it accelerated the apoptosis of tumor cells under laser irradiation. Importantly, *in vivo* experiments demonstrated that HPAA/GO-RGD@DOX, when combined with laser irradiation, effectively targeted the tumor site and inhibited tumor growth. In conclusion, HPAA/GO-RGD@DOX exhibits high tumor targeting efficiency and a strong photothermal effect. This study suggests that the HPAA/GO-RGD@DOX composite holds great promise as a new type of functional 2D nanoparticle with photothermal capabilities for breast cancer therapy.

## Graphical abstract

**Figure:**
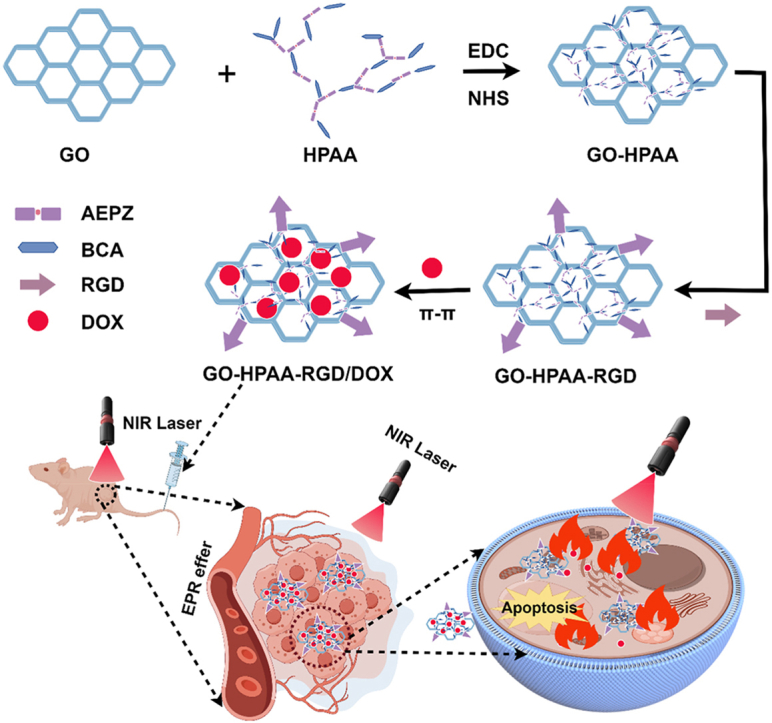

## 1 Introduction

Breast cancer is the most prevalent cancer worldwide, posing a significant threat to the lives and health of patients. In China alone, the annual number of new cases of breast cancer has reached 420,000, with 120,000 deaths [1]. While the 5-year survival rate of breast cancer patients in China has notably improved over the past decade, there still remains a considerable gap compared to European and American countries [2]. Despite advancements in early diagnosis and treatment, recurrence and metastasis of breast cancer present challenges in subsequent treatment lines, including drug resistance, with a 5-year survival rate of less than 25 % for affected patients. Given these circumstances, there is an increasing need to explore effective therapeutic strategies aimed at improving the survival rates and quality of life for breast cancer patients.

At present, the treatment of breast cancer mainly includes chemotherapy, radiation and surgery, with not-so-effective results accompanied by grievous side effects [3]. Recent advancements in nanotechnology and materials science have introduced promising opportunities for the development of photothermal therapy (PTT) in breast cancer treatment [4]. The development of nanotechnology has opened up new avenues for photothermal therapy (PTT) for breast cancer treatment. PTT is a strategy using near infrared (NIR) light irradiation and photothermal materials to generate hyperthermia for tumor ablation [5,6].

Various nanomaterials that absorb NIR light, such as gold nanoparticles Carbon nanotubes [7,8] and graphene [9,10] have been studied for PTT treatment of tumors with high therapeutic efficiency. Among these nanomaterials, 2D dimensional nanoparticles graphene oxide (GO) has been widely used as a photothermal agent for the treatment of tumors due to its large specific surface area, strong near-infrared absorption and good biocompatibility [11]. The π-π accumulation of surface-rich oxygen-containing groups on graphene oxide makes it an ideal chemotherapeutic vehicle for loading hydrophilic and hydrophobic drugs [[12], [13], [14]]. The cytotoxicity of graphene oxide nanosheets correlates with their transverse size and degree of oxidation, with the higher the degree of oxidation, the smaller the graphene oxide, and the lower the cytotoxicity [14]. However, it has been found that while photothermal treatment kills tumors, it also causes significant damage to normal tissues and cells.

To enhance the effectiveness of photothermal therapy while minimizing damage to normal cells and tissues, we conducted targeted modifications of photothermal drug carrier materials. Integrin αvβ3 is a cell adhesion receptor crucial for the formation, survival, and maturation of new blood vessels during angiogenesis [15,16]. Although its expression levels are low in non-malignant and benign diseases, it is widely expressed in breast cancer [17,18]. The expression level of αvβ3 is correlated with tumor invasiveness, making it an essential biological target for molecular imaging probes in the early diagnosis of tumors [19]. Synthetic peptides derived from the arginine-glycine-L-Aspartic Acid (RGD) sequence are well-known regulators of cell adhesion and can bind integrin αvβ3 with high affinity and specificity [[20], [21], [22]]. Consequently, radiolabeled monomers and polycyclic RGD peptides have been developed for imaging of integrin αvβ3-positive cancers.

**Scheme 1:**
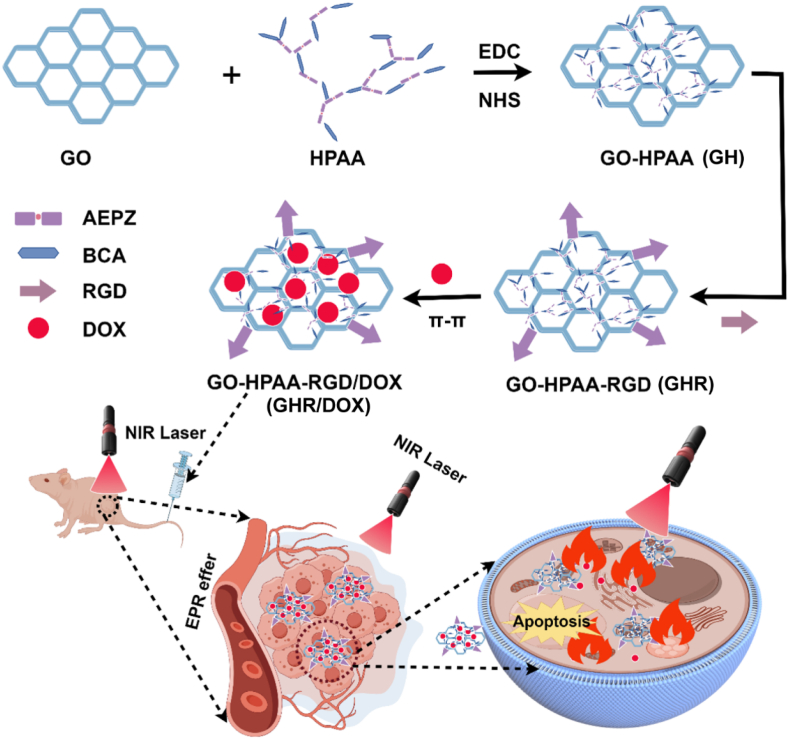
Schematic illustration of the RGD targeting combinational chemotherapy and PTT against breast cancer.

In this work, we designed an RGD-targeted multifunctional chemotherapy combined with photothermal therapy nanodrug delivery system base Graphene Oxide (GO) nanoparticles (Scheme 1). The nanocarriers demonstrated excellent photothermal effects and stability under NIR illumination. Moreover, *in vitro* experiments revealed that the composite nanocarriers exhibited good biocompatibility, blood compatibility, and an outstanding synergistic anti-tumor effect combining chemotherapy with photothermal therapy, along with targeted delivery capability. *In vivo* animal experiments demonstrated that RGD-modified nanocarriers exhibited enhanced accumulation at tumor sites and demonstrated superior synergistic anti-tumor effects.

## 2 Materials and experimental

### 2.1 Materials

DOX·HCl, Cysteamine dihydrochloride, N-hydroxysuccinimide (NHS), and 1-ethyl-3-(3-dimethyl amino-propyl) carbodiimide hydrochloride (EDC) were purchased from Aladdin Biochemical Technology Co., LTD (Shanghai, China). The RGD peptide (98 %) was obtained from Beijing Jiankai Technology Co., LTD (Beijing, China). Cyanine 5.5 (Cy5.5) was obtained from Thermo Fisher Scientific (Waltham, USA). 4′,6-diamidino-2-phenylindole (DAPI), Live and Dead Cell Activity/Toxicity Detection Kit and Annexin FITC/V-PE were obtained from Sigma-Aldrich (St. Louis, USA). The mouse epithelioid fibroblasts cell line (L929) was obtained from Guangzhou Beogyne Biotechnology Co., LTD (Guangzhou, China). The human breast carcinoma cells (MCF-7) were obtained from Guangzhou First Military Area Hospital (Guangzhou, China). Both L929 and MCF-7 cells were cultured in DMEM medium (Gibco, Life Technologies, USA) containing 10 % (v/v) fetal bovine serum (FBS) and 1 % (v/v) penicillin/streptomycin. The cells were incubated at 37 °C under an atmosphere of 5 % CO_2_.

### 2.2 Synthesis and characterization of GO-HPAA-RGD (GHR)

Firstly, N, N-bis(acryloyl) cystamine (BAC) was synthesized according to our previous work [23]. Briefly, 2.50 g cysteamine dihydrochloride was dissolved in 11 mL pure water, followed by 3.30 mL acryl chloride dissolved in 3.30 mL dichloromethane and 1.76 g sodium hydroxide dissolved in 4.40 mL water. The two solutions were added alternately to cysteamine dihydrochloride solution drop by drop in an ice bath and quickly stirred. After the addition, the ice bath was removed and the reaction solution was kept at room temperature for 6 h. After the reaction is complete, 10 times the volume of dichloromethane is added to extract it and cleaned with pure water for 3 times. Finally, the solvent dichloromethane was removed by rotary evaporation at 45 °C, and the white powdered BAC product was obtained by vacuum drying overnight. Next, hyperbranched polyamides (HPAA) were prepared by Michael addition reaction of BAC with piperazine (AEPZ). Specifically, prepare 10 mL of pure water and methanol mixture at room temperature (the volume ratio of pure water to methanol is 1/3), add 0.222 g of calcium chloride, then add another 0.64 g of BAC to it, and stir until it is completely dissolved. Then 160 μL AEPZ was dissolved in 300 μL of methanol and added into the reaction solution. Then, condense and reflux reaction at 50 °C for 48 h, and add 320 μL AEPZ for another 12 h. After the reaction, the product was collected and its pH was adjusted to 4–6 with hydrochloric acid. Then the product was dialyzed in pure water for 2 days (MWCO = 1000) and freeze-dried to obtain the final white floc product HPAA.

For the GO conjugation, carboxylated GO nanosheets were synthesized by adding 1.2g of sodium hydroxide and 1.0g of chloroacetic acid into the GO suspension (10 mL, 1 mg/mL), and the reaction was carried out in an ice bath. After ultrasonic treatment for 3 h, the reaction was stirred overnight. The carboxylated GO nanosheets were obtained by adjusting the pH to neutral with hydrochloric acid, centrifugal washing at 8000 rpm for 10 min for 2–3 times, and dialysis with deionized water for one day. Then, HPAA with a mass ratio of 1:4 was added for a 2-h stirring reaction at room temperature, and then an appropriate amount of EDC and NHS were added for an overnight stirring reaction at room temperature. Adjust pH to neutral with hydrochloric acid, perform centrifugal washing, and dialysis for a day. The GO-HPAA (GH) nanosheet material was obtained.

The RGD peptide was conjugated to GH through the Michael addition reaction between the sulfhydryl group in the RGD peptide and the maleimide moiety in HPAA. Briefly, 10 mg of RGD peptide was dissolved in 6 mL of water, and 15 mg of EDC and 12 mg of NHS were added to the RGD solution and stirred for 4 h. The RGD solution was added drop by drop into 15 mL of GH solution and stirred overnight at room temperature. At the end of the reaction, the excess RGD polypeptide was removed by centrifugation at 4000 rpm for 30 min at 4 °C and washed twice to obtain GHR.

The chemical structures of the BCA and HPAA were confirmed by proton nuclear magnetic resonance spectroscopy (^1^H NMR, Bruker-500, Bruker, Germany). The morphology and microstructure were observed by TEM (Tecnai G20, USA). The size and zeta potential of nanoparticles were measured by Zetasizer Nano ZS detector (Malvern Instruments, Worcestershire, UK). The components in the samples GO, HPAA, and GH were determined by thermogravimetric analysis (NETZSCH, TG209F3-ASC, Germany).

### 2.3 Preparation and characterization of drug-loaded nanomaterials

GHR nanoparticles containing DOX were prepared by self-assembly using the dialysis method. In detail, 40 mg GHR and 8 mg DOX were dissolved in 4 mL DMSO, and an appropriate amount of triethylamine was added to neutralize the hydrochloric acid in DOX hydrochloride. The solution was stirred magnetically at room temperature (25–30 °C) for 12 h, and then dropped into 40 mL deionized water. DMSO and unencapsulated DOX were removed by dialysis with 2 kDa MWCO after 12h of stirring in the dark. During the process, the water was changed at least 5 times, and the GHR/DOX were obtained by freeze-drying. The DOX content was determined using a UV–visible (UV–vis) spectrophotometer (UV1800, Hitachi, Japan) at 488 nm. Specifically, a series of DOX solutions with different concentrations of 250, 125, 62.5, 31.25, 15.6, 7.8, 3.9 μg/mL were prepared, and their absorbance at 488 nm was measured. The standard curve equation Y = 0.012x +0.0829 was obtained, and the drug loading rate was calculated to be 20.5 %. To further verify successful drug loading, DOX, GHR, and GHR/DOX were characterized using UV spectroscopy. To investigate the *in vitro* release of DOX, DOX-loaded nanoparticles were placed in a PBS system (pH = 7.4 or pH = 5.5). Specifically, two copies of GHR/DOX were dispersed into 1 mL of PBS containing 10 % Tween-80, respectively, and incubated in a 37 °C constant temperature shaker. The release was assessed at different time intervals (0.5, 1, 3, 5, 20, 24, 48, and 72 h, with additional illumination for one group), followed by replenishing with fresh PBS to continue the oscillating incubation. The amount of DOX released was calculated based on the standard release curve, and then the cumulative release amount was calculated.

### 2.4 The photothermal performance of the particles

To evaluate the photothermal performance of materials at different concentrations, GHR was dispersed in water to prepare solutions with varying concentrations (0, 62.5, 125, 250, 500 μg/mL). At room temperature, 1 mL of the above-mentioned solutions with different concentrations were added to a colorimeter, and a thermocouple thermometer was inserted. The liquid surface was then irradiated with 808 nm near-infrared light at 1.5 W/cm^2^ for 5 min, and the temperature was recorded every 10 s. At the same time, deionized water was used as control group and recorded by infrared thermal imager. Additionally, the influence of laser power on the photothermal effect was investigated. Specifically, 1 mL of GHR solution with a concentration of 250 μg/mL was added to a colorimetric dish. The liquid surface was irradiated with 808 nm near-infrared light at different powers (1, 1.5, and 2 W/cm^2^) for 5 min. At room temperature, a thermocouple thermometer was inserted into the liquid level and the temperature was recorded every 10 s. Pure water was used as the control group. Finally, the photothermal stability of the material was monitored. 1 mL GHR solution with a concentration of 250 μg/mL was added to the colorimeter, and the power was 1.5 W/cm^2^. Near-infrared light at 808 nm was irradiated for 5 min, after which the irradiation was stopped to allow the temperature to return to its initial level. This process was repeated 5 times, with temperature recorded every 10 s at room temperature. The photothermal efficiency was calculate according to the previous method [24].

### 2.5 Blood compatibility

#### 2.5.1 Red blood cell morphology

Initially, fresh whole blood was centrifuged at 1000×*g* for 5 min, and the underlying red blood cells (RBC) were collected. Subsequently, four concentrations of GHR (0.5 mg/mL, 0.2 mg/mL, 0.1 mg/mL and 0.01 mg/mL) were configured, and PBS was used as control group. Each sample solution was mixed with an appropriate amount of red blood cells, incubated for 1 h. The supernatant was discarded by centrifugation, and the resulting precipitate containing RBCs was washed with PBS and subsequently fixed with 4 % paraformaldehyde for 1 h. After fixation, the RBCs were dehydrated with 70 %, 85 %, 95 % and 100 % ethanol for 10 min each. After natural air drying, the samples were sprayed with gold, and the changes of red blood cell morphology were observed by scanning electron microscope (SEM).

#### 2.5.2 Hemolysis analysis

The PBS solution with different concentrations of GHR (0.5 mg/mL, 0.2 mg/mL, 0.1 mg/mL and 0.01 mg/mL) was configured. Deionized water and PBS were used as positive and negative controls, respectively. 4 mL samples of each concentration were collected in centrifuge tubes, and 200 μl of 16 % red blood cell suspension was added and incubated together. The supernatant was collected by centrifugation at 1000×*g* for 5 min at the present time points (0.5 h, 1 h, 3 h, 5 h, 7 h and 24 h). The sample was measured by microplate analyzer at 540 nm, and the hemolysis rate was calculated by the following formula:

Hemolysis rate (%) = (A-C)/(B-C) × 100

Where A, B and C represent the absorbance of the experimental group, positive control group and negative control group respectively (each sample was made in parallel with three groups).

#### 2.5.3 Activated partial thromboplastin time (APTT) and prothrombin time (PT) assay

Fresh anticoagulated whole blood was centrifuged at 1000×*g* for 10 min, and the resulting supernatant (platelet-poor plasma) was collected. Platelet-poor plasma (270 μL) was mixed with different concentrations of GHR (30 μL in PBS). After the appropriate reagents were added, APTT and PT of the samples were measured with an automated coagulation analyzer (STAR Evolution, Diagnostica Stago, Assiernes, France).

### 2.6 *In vitro* cytotoxicity study

The cytotoxicity of GHR on L929 cells was evaluated by CCK-8 assay. Firstly, L929 cells were seeded in 96-well plates at a density of 5000 cells/well and incubated overnight in a CO_2_. Subsequently, the original medium was aspirated and replaced with fresh complete medium containing different concentrations of GHR. The concentration range of the GHR was 10–500 μg/mL, with 5 parallel concentrations for each concentration. The cells were cultured in an incubator for 24 h. After culture, the cells were washed once with PBS and 100 μl of fresh medium (containing 10 % CCK-8) was added to each well. Finally, the absorbance at 450 nm was detected and recorded by microplate reader.

Moreover, previous studies have demonstrated that GO has good photothermal properties, and the temperature can rise sharply in a short time under NIR near-infrared laser irradiation, which has important application value for the photothermal treatment of cancer cells [25]. To investigate the cytotoxicity of GO nano-drug carrier under anticancer drugs and photothermal conditions, MCF-7 cells were selected for this study. The following nanoparticles were added to each group of cells for co-culture: DOX, GHR + NIR, GHR/DOX and GHR/DOX + NIR were used as control group, where NIR represented the treatment with 808 nm NIR near infrared laser irradiation, laser power density was 1.5 W/cm^2^, illumination time was 5 min. The concentrations of DOX ranged from 1 to 40 μg/mL. Cells in each group were co-cultured for 24 h under the same conditions, and the cell activity was detected by CCK-8. Additionally, the live/dead cell staining kit was employed to visually depict the effect of photothermal drugs combined with anti-tumor drugs in MCF-7 cells. Live and dead cells were stained with calcein-AM and PI, respectively, and imaged using an inverted fluorescence microscope (Olympus, Tokyo, Japan).

### 2.7 Cell apoptosis

MCF-7 cells were seeded in 24-well plates at a density of 5 × 10^4^ cells/well and cultured overnight. Then the original medium was aspirated and complete medium containing PBS, GHR, DOX and GHR/DOX was added. A part of the medium was irradiated with an 808 nm NIR laser at 1.5 W/cm^2^ for 5 min and incubated for 24 h. At the conclusion of the experiment, the cells were washed with PBS, trypsinized, and the supernatant was removed by centrifugation. The cells were then treated with the Annexin V-APC Apoptosis Detection Kit (BD Biosciences, USA) and subsequently analyzed by flow cytometry.

### 2.8 Cell endocytosis and targeting assay

The ability of targeted peptides to target cells was evaluated by endocytosis assay. The specific steps were summarized as follows: First, MCF-7 cells were seeded in 24-well plates at a density of 5 × 10^4^ cells per well and incubated overnight. Then, fresh medium containing GH-FITC and GHR-FITC complexes was added to incubate the cells for 2 h and 4 h, respectively. The final concentration of GO in each well was 40 μg/mL. After incubation, each well was washed three times with PBS to remove free complexes, digested with trypsin, and harvested by centrifugation. Finally, cells were resuspended with an appropriate amount of PBS, detected by FCM, and data were analyzed by Flow Jo7.6.1 software.

The cellular uptake and intracellular distribution of GH-FITC and GHR-FITC nanoparticles in MCF-7 cells were visualized using confocal microscopy. Specifically, MCF-7 cells were seeded at a density of 2 × 10^5^ cells/dish and incubated for 12 h. Then, the old medium was removed and fresh complete medium containing FITC-labeled GHR and GH complexes was added. After 2 h of incubation, the cells were washed with PBS and fixed with 4 % paraformaldehyde. After three washes with PBS, 1 μL of DAPI was added to each dish to stain the nuclei for 15 min. Finally, the cells were washed three times and 2 mL of PBS was added to each dish to maintain the normal morphology of the cells. The treated cells were observed and photographed using a laser confocal microscope.

### 2.9 *In vivo* antitumor effect

The SPF BALB/C nude mice (3–4 weeks old, female) were purchased from Guangdong Medical Laboratory Animal Center. The experimental animal experiments were approved by the Institutional Animal Care and Use Committee (IACUC) of Ruige Biotechnology and followed the relevant laws and guidelines. The steps to establish the mouse tumor model were as follows: MCF-7 cells were digested with trypsin, centrifuged, and resuspended in PBS. An equal volume of high-concentration stromal gel was added. The cells were seeded into the axilla of the right forelimb of BALB/C nude mice at a cell density of 2 × 10^6^ cells/100 μL, and the tumor size was observed daily. When the tumor volume reached about 100 mm^3^, the mice underwent further treatment. The nude mice of the tumor model were randomly divided into 6 groups: PBS, PBS + NIR, DOX, GHR/DOX, GHR + NIR, GHR/DOX + NIR. The DOX was administered at a concentration of 4 mg/kg every two days for treatment duration of 14 days. Tumor volume and body weight of mice were measured every two days. After 14 days, all mice were euthanized, tumors were collected, weighed, and photographed. Subsequently, the tumors were fixed with tissue fixation solution, embedded, and evaluated for antitumor effects through hematoxylin and eosin (H&E) staining, terminal transferase labeling (TUNEL) analysis, and immunohistochemical Ki67 and CD31 analysis.

To evaluate the i*n vivo* thermal imaging effect of the materials, tumor-bearing mice were injected intravenously with PBS, GHR, and GH. The mice were irradiated with an 808 nm laser at 1.5 W/cm^2^ for 5 min, and infrared thermal images were captured. The tumor-targeting ability of GHR was evaluated using fluorescence imaging in mice with a small animal living imager. Initially, two tumor-bearing nude mice with tumors of approximately 100 mm^3^ were selected. Subsequently, 100 μL of GHR-FITC and GH-FITC were injected into the tail vein, respectively (GO volume: 20 mg/kg). Fluorescence imaging was performed on the nude mice. The heart, liver, spleen, lung, kidney and tumor tissues were removed for ex vivo imaging. The excitation wavelength is 460 nm–550 nm and the emission wavelength is 520 nm–530 nm.

### 2.10 *In vivo* safety assessment

After the mice were euthanized, the major organs of the heart, liver, spleen, lungs and kidneys were collected, immersed in tissue fixative, and subjected to routine paraffin embedding and tissue sectioning. Sections were then stained with hematoxylin and eosin and photographed using a fluorescence microscope. Serum biochemical indexes were analyzed 14 days after treatment. Blood was collected from the eye socket and allowed to stand at room temperature. Then the upper layer of serum was collected by centrifugation (3000 rmp, 5 min), and serum biochemical indexes including albumin (ALB), lactate dehydrogenase (LDH), urea nitrogen (BUN), creatinine (CRE), total cholesterol (T-cho), serum total protein (TP), low-density lipoprotein (LDL), globulin (GLB), and glucose (GLU) were analyzed.

### 2.11 Statistical analysis

All measurements were performed at least three times and all data were presented as mean ± standard deviation (SD). Student's t-tests were used in the analysis of two-group parameters. ∗P < 0.05 were considered statistically significant.

## 3 Results and discussion

### 3.1 Synthesis and characterization of GHR

**Fig. 1:**
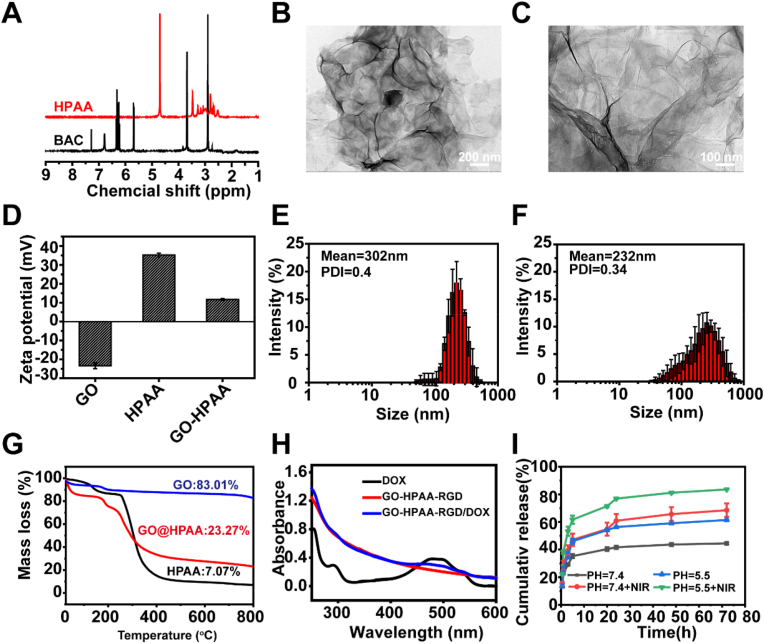
(A) The ^1^H NRM of the BAC and HPAA; TEM of (B) GO and (C) GH; (D) Zeta potential of GO, HPAA and GH; Hydrodynamic diameter of the (E) GO and (F) GH; (G) TG curves of GO, HPAA and GH; (H) UV–vis absorbance spectra of DOX, GHR and GHR/DOX; (I) DOX release profiles of GHR@DOX at pH 7.4 and pH 5.5, with or without NIR irradiation (808 nm, 1.5 W/cm^2^).

In this study, we synthesized 2D-dimensional nanoparticles for targeted treatment of breast cancer by combining photothermal therapy and chemotherapy. The synthesis route of HPAA/GO-RGD@DOX is illustrated in Scheme 1. Firstly, BAC was synthesized by amidation reaction between cystamine dihydrochloride and acryloyl chloride Subsequently, HPAA was synthesized by Michael addition polymerization between BAC and AEPZ. The ^1^H NMR spectrum of BCA and HPAA was shown in Fig. 1A. It was found that HPAA characterized peaks at 3.43 (m, 4H, CONHCH_2_CH_2_S), 3.09 (m, 4H, NCH_2_CHCO), 3.04 (m, 4H, NCH_2_CH_2_CO), 2.78 (m, 4H, CONHCH_2_CH_2_S) and 2.3–2.7 (m, 12H, CH_2_ from AEPZ). These results indicated that HPAA was synthesized successfully [23]. Secondly, GO was conjugated with HPAA via electrostatic adsorption. The morphology, zeta potential and diameter were detection by TEM and DLS. As shown in Figure (1B, C)**,** the GO exhibited a single-layered tulle-like structure with a diameter of approximately 400–600 nm, and no significant change was observed after composite with HPAA. From Figure (1E, F), the DLS results shown that the particle size of GO and GH was 302 ± 12 nm and 232 ± 23 nm, respectively. The reduction in particle size of GH may be attributed to the effect of electrostatic adsorption, resulting in closer linkage [26]. In addition, we also evaluated its particle size stability under different physiological conditions (PBS or 10 % FBS). As shown in Fig. S1, GH has no obvious aggregation under different physiological conditions, and the particle size in 10 % FBS is slightly larger, which may be due to a small amount of protein adsorption [27]. Additionally, as shown in Fig. 1D, the zeta potentials of GO, HPAA, and GH were −24.6 mV, 35.2 mV, and 10.7 mV, respectively. This affirmed that HPAA was combined with GO through electrostatic adsorption. Subsequently, the composition of GH was further characterized using TG. As shown in Fig. 1G,the contents of GO and HPAA in GH nanomaterials are 23 % and 77 %, respectively. In addition, DOX, GHR and GHR/DOX were characterized by UV spectrophotometer. As shown in Fig. 1H, the characteristic absorption peak of DOX is at 250 nm and 480 nm. Moreover, the same position absorption peak of GHR/DOX was observed, which indicated that DOX was successful loaded. To evaluate the drug release behavior of HPAA/GO-RGD@DOX, we conducted *in vitro* drug release studies under physiological (pH 7.4) and acidic conditions (pH 5.5), with and without NIR irradiation. The acidic condition (pH 5.5) was selected to simulate the slightly acidic tumor microenvironment. As shown in Fig. 1I, the cumulative release of DOX at pH 5.5 reached approximately 65 % within 24 h, significantly higher than that at pH 7.4 (∼40 %).

In addition, under pH 7.4 with NIR irradiation, the 72 h cumulative release increased to 68 %, indicating that NIR irradiation effectively accelerated drug release. Similarly, under pH 5.5 with NIR irradiation, the cumulative release further increased to 83.5 % within 72 h, demonstrating the synergistic effect of acidic pH and photothermal stimulation in promoting DOX release [28,29].

### 3.2 Photothermal effect *in vitro*

**Fig. 2:**
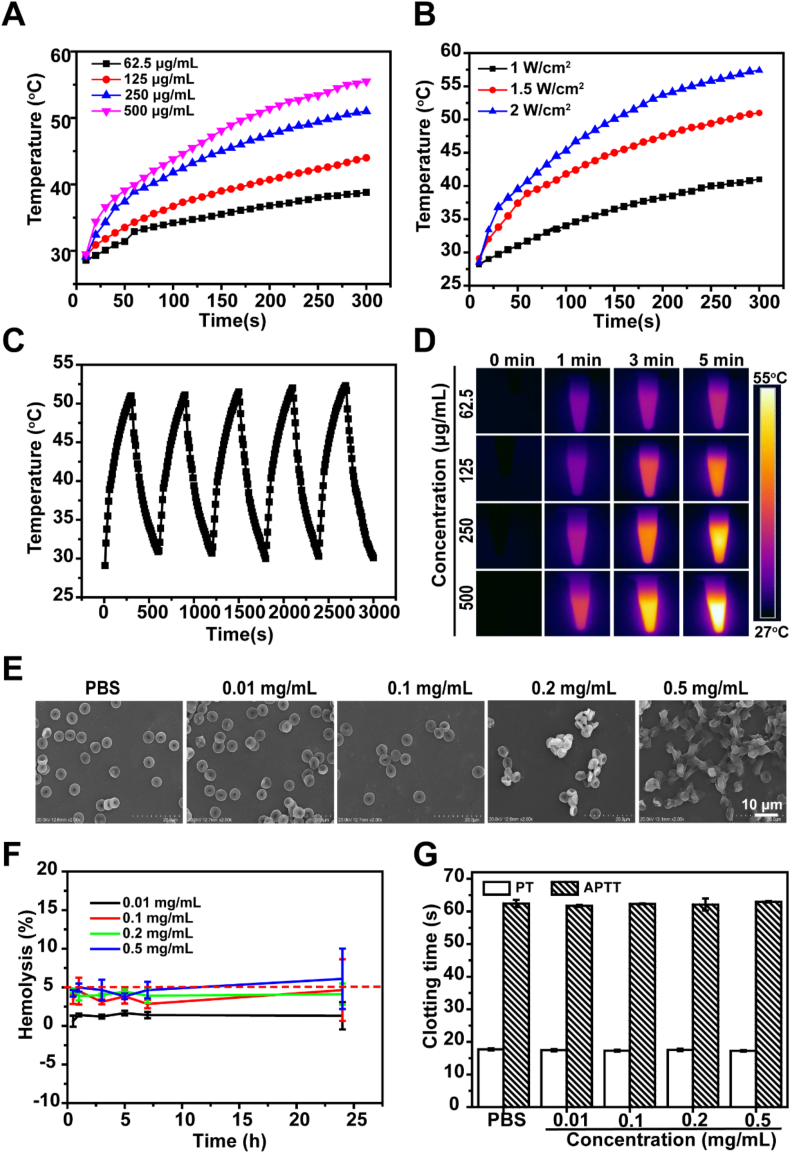
(A) Temperature curve of different concentrations of GHR solution under NIR; (B) Temperature of the GHR solution at different laser power density; (C) Temperature rise and cooling cycles of GHR irradiated by NIR switch. The irradiation conditions are all: 808 nm NIR and 1.5 W/cm^2^ and (D) Thermal imaging of different concentration of GHR solution under NIR; (E) Red blood cell morphology of GHR; (F) Hemolysis of GHR and (F) APTT and PT assay of GHR.

We further investigated the photothermal performance of GHR using 808 nm laser. Fig. 2A shows the time-temperature curve of the material under different concentration conditions. It is evident that as the concentration increases, the heating rate of each GHR sample also increases. Notably, when the material concentration reached 500 μg/mL, the temperature surged to 57 °C within 5 min. Additionally, we also investigated the photothermal properties of the nanoparticles under different power conditions. As shown in Fig. 2B, with the increase of the laser power, the temperature of the GHR rises faster and the maximum temperature increases. Specifically, at 1.5 W/cm^2^, the temperature increased from 25 °C to 52 °C, while at 2 W/cm^2^, the temperature increased from the initial 25 °C–58 °C. Furthermore, we assessed the photothermal stability of the material. As shown in Fig. 2C, the GHR exhibited excellent photothermal stability. The GHR nanosystem exhibited a photothermal conversion efficiency of 39.68 % under 808 nm NIR laser irradiation. Their photothermal conversion efficiency remains consistent through five consecutive heating-cooling cycles. The infrared thermal image under 808 nm laser irradiation, from Fig. 2D, which demonstrates that the temperature of the nano-system can rise rapidly within the irradiation time, and the increasing degree is positively correlated with the material concentration. All above experimental results proved that the successful synthesis of GHR with excellent photothermal properties.

### 3.3 Blood compatibility analysis

*In vivo* administration, nanoparticles will inevitably come into contact with blood, so the evaluation of its blood compatibility is particularly important [30]. We first examined the effect of different concentrations of GHR on erythrocyte aggregation and morphology by SEM. As shown in Fig. 2E, compared with the PBS negative control group, erythrocytes in the experimental groups with different concentrations of GHR did not aggregate and their morphology was basically normal, indicating that the concentration of GHR below 0.2 mg/mL had no effect on erythrocytes. Additionally, the extent of membrane damage by GHR by erythrocyte hemolysis *in vitro*. The higher the hemolysis rate, the greater the damage of the material to the erythrocyte membrane. Generally speaking, a hemolysis rate exceeding 5 % is considered significant hemolysis [31]. As observed in Fig. 2F, the hemolysis rate of GHR was less than 5 % at each incubation time point, except at a concentration of 0.2 mg/mL, this indicates that GHR does not cause significant damage to the erythrocyte membrane even at a concentration of 0.2 mg/mL. APTT and PT are routine parameters for plasma coagulation in clinic. Because the formation pathway of prothrombin activator is different from that of blood coagulation factor, the blood coagulation cascade includes endogenous pathway, exogenous pathway and common pathway [32]. APTT measures intrinsic pathway performance and refers to the time it takes for an fibrine thrombus to form in plasma after the addition of partial thrombin reagent and CaCl_2_. PT measures extrinsic pathway performance and refers to the time it takes to form fibrin clots after the addition of tissue thromboplastin. The effect of GHR on APTT and PT is shown in Fig. 2G. Compared with PBS group, different concentrations of GHR had no significant effect on APTT and PT. The results of erythrocyte morphology, hemolysis APTT and PT demonstrate that GHR exhibits good blood compatibility and holds potential in clinical practice.

### 3.4 Biosafety evaluation *in vitro*

**Fig. 3:**
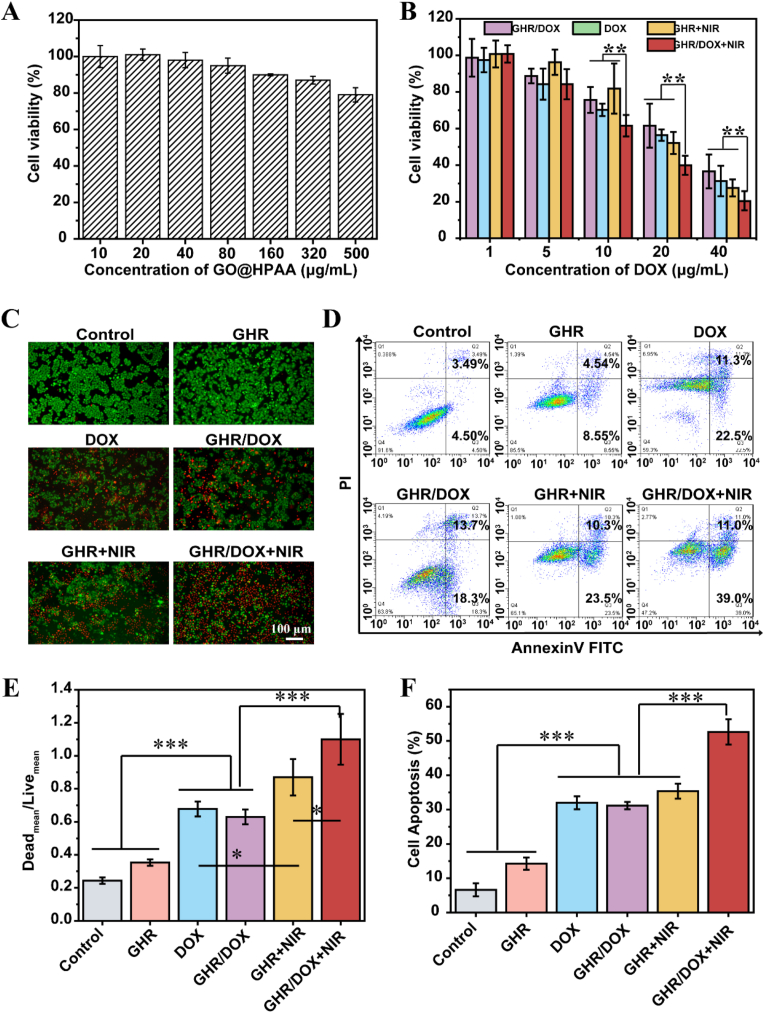
(A) *In vitro* cytotoxicity of GHR on L929 cells over an evaluation period of 24 h. (B) CCK-8 assay of MCF-7 cells viability after treatment with the GHR/DOX, GHR + NIR and GHR/DOX + NIR; (C) Images of PI and Calcein-AM double-stained MCF-7 cells under different treatments; (D) Flow cytometry analysis of MCF-7 cell apoptosis in different treatment groups; (E) Quantification of live-dead fluorescence ratio of MCF-7 cells after different treatments; (F) Quantitative detection of MCF-7 cells after different treatments using flow cytometry analysis; the data are shown as mean ± SD (n = 3).

For detecting the biocompatibility of GHR, the cytotoxicity on L929 cells was evaluated by CCK-8 assay. Fig. 3A shows the survival rates of L929 cells after 24 of co-culture with GHR. The results indicate that the cytotoxicity of GHR was not significantly different from that of the control group. When the concentration of HPAA reached 500ug/ml, the cell survival rate was still about 80 %, indicating which have good biocompatibility.

### 3.5 Evaluation of antitumor effect *in vitro*

To assess the synergistic effects of GHR Nanoparticles PTT/chemotherapy combination treatment against MCF-7 Cells. The cytotoxicity of different treatments was detected by CCK-8 assay and the results are shown in Fig. 3B. At low concentrations, DOX, GHR/DOX, GHR + NIR and GHR/DOX + NIR had no obvious cytotoxicity. However, with increasing concentration, the cytotoxicity of GHR/DOX and GHR/DOX + NIR nanoparticles significantly intensified at the same DOX concentration. Moreover, it is evident that at concentrations below 10 μg/mL, GHR/DOX + NIR demonstrated the highest cytotoxicity, followed by GHR/DOX, while GHR + NIR exhibited the least toxicity. This indicates that at low concentration, DOX released from nanomaterials serves as the primary anti-tumor agent, and the photothermal effect at low concentration is not obvious. Conversely, at concentrations exceeding 10 μg/mL, the cytotoxicity of GHR/DOX + NIR was the highest, followed by GHR + NIR, whereas GHR/DOX exhibited the least cytotoxicity. Conversely, at concentrations exceeding 10 μg/mL, the cytotoxicity of GHR/DOX + NIR was the highest, followed by GHR + NIR, whereas GHR/DOX exhibited the least cytotoxicity.

Next, we further visualized the synergistic antitumor effects using the live/dead cell staining kit. As shown in Fig. 3C and Fig. S2A, there was almost no red fluorescence in cells either solely irradiated by NIR or treated with GHR, which was not significantly different from the control group, indicating that NIR irradiation and GHR would not cause cell damage. However, after the intervention of the DOX, GHR + NIR, GHR/DOX, and GHR/DOX + NIR groups, the cells exhibited obvious red fluorescence, indicating significant induction of cell death. In addition, we quantified the ratio of green (live cells) and red (dead cells) fluorescence intensities in each group (Fig. 3E). It can be seen that GHR/DOX + NIR had a higher ratio than DOX, GHR + NIR, and GHR/DOX, further confirming the synergistic effect of PTT and chemotherapy, which is similar to the results of CCK-8 assay.

Subsequently, we evaluated the apoptosis of MCF-7 cells under different treatments, and the flow cytometry results and statistical results are shown in Fig. 3D–S2B and 3F. Laser irradiation alone had little effect on apoptosis (7.88 %). In the absence of laser irradiation, GHR had no significant induction of apoptosis (13.9 %), while GHR/DOX nanoparticles induced apoptosis significantly (31.16 %), indicating that some DOX had been released from the nanoparticles. However, under laser irradiation, the nanoparticles in the GHR/DOX + NIR group had the strongest apoptosis-inducing effect, with a normal cell apoptosis rate of 52.8 %. In addition, the apoptosis rates of the DOX, GHR/DOX, and GHR + NIR groups were 31.98 %, 31.16 %, and 35.3 %, respectively. Therefore, we concluded that GHR/DOX + NIR nanoparticles can induce more apoptosis in MCF-7 cells through PTT combined with chemotherapy.

### 3.6 Cellular uptake and targeting assay

**Fig. 4:**
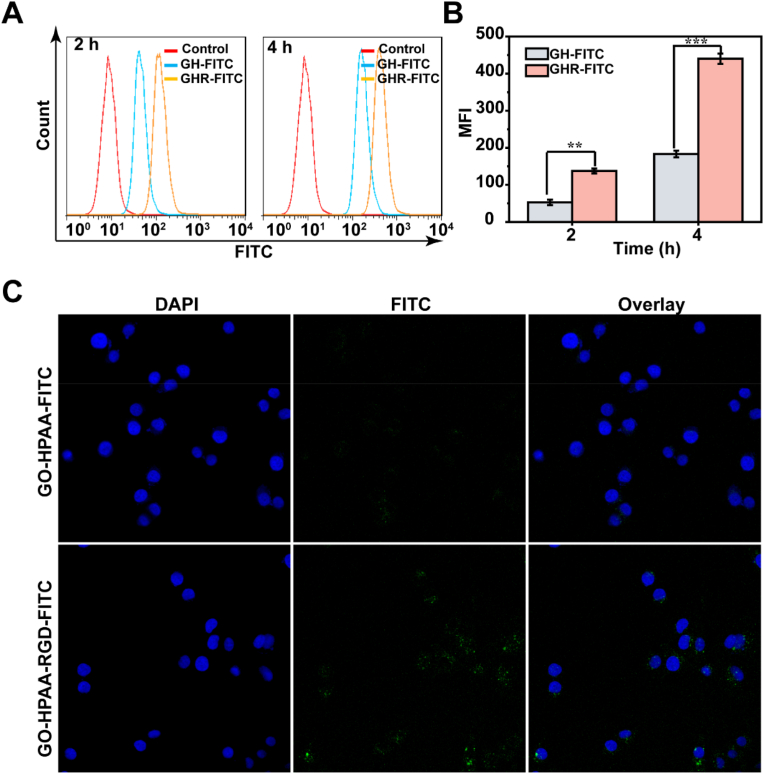
(A) Flow cytometry analysis of fluorescence peak in MCF-7 cells incubated with GH or GHR at different time; (B) Cellular uptake evaluation of GH or GHR using CLSM over an incubation period of 2 h.

The endocytosis and specific targeting of nanomaterials are shown in Fig. 4. The ability of RGD targeting was verified by quantitative analysis the intensity of fluorescence in the cells. As shown in Fig. 4A and B, at the same time, the intensity of GHR was higher than GH, and the difference was most obvious at 4 h. Moreover, the fluorescence intensity of both groups increased with time, but the fluorescence intensity of GHR remained higher than that of GH. These results indicate that RGD significantly enhances the targeting effect of the nano-system. This is because integrin αvβ3 is a common over-expression cell adhesion molecule in tumor vessels and some cancer cells, and plays a key role in tumor angiogenesis and cancer cell migration, invasion and metastasis. RGD-targeted peptide modification can provide specific interactions between nanoparticles and cancer cells, thus affecting tumor accumulation, cellular internalization, and intracellular localization of nanoparticles [33,34]. Furthermore, Confocal Laser Scanning Microscopy (CLSM) was employed to analyze endocytosis. As shown in Fig. 4C, GHR enters the cell, disrupting lysosome integrity and resulting in numerous green spots near the nucleus. This means that GHR can escape from the lysosome after endocytosis and subsequently migrate to the nucleus, facilitating drug release.

### 3.7 Evaluation of antitumor *in vivo*

**Fig. 5:**
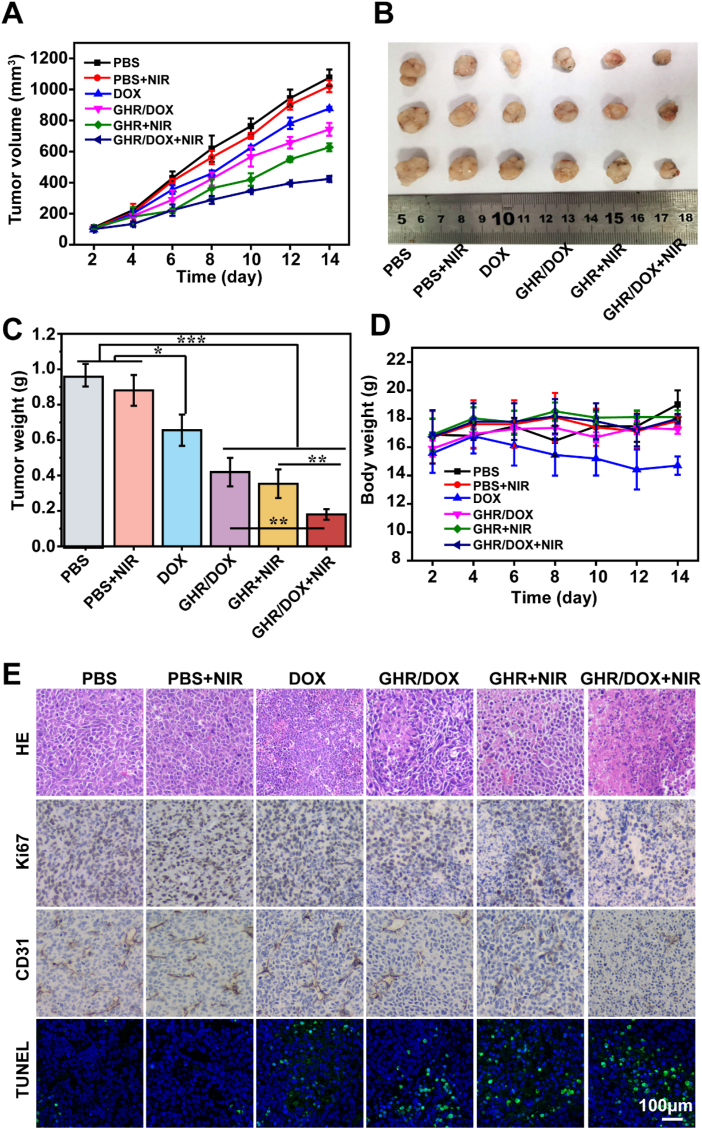
(A) *In vivo* tumor growth curves of MCF-7 tumor-bearing mice treated with different formulations. (B) Representative image of MCF-7 tumors at the 14th day. (C) The tumor weights excised from different groups after 14 days treatment. (D) Body weight changes of mice treated with different formulations during the treatment. (E) Immunohistochemical analyses of H&E, TUNEL, CD31 and Ki67 for MCF-7 tumor tissues after the last treatment with different formulations *in vivo*.

On the basis of cell level *in vitro*, we further investigated the PTT and chemotherapy therapy effect of GHR/DOX on MCF -7 breast cancer tumor model *in vivo*. The therapy effect was evaluated by the determination of tumor volume and weight, and the results are shown in Fig. 5A–C. In the PBS control group, tumor volume sharply increased to 1100 mm^3^ within 14 days. Similarly, the PBS + NIR group exhibited a tumor growth trend similar to that of the PBS group. The effect of DOX alone was not significant, with the tumor volume still at 850 mm^3^ at 14 days. In contrast, both the GHR/DOX and GHR + NIR groups demonstrated certain anti-tumor effects, with the tumor volume being almost half that of the PBS group. Notably, the GHR/DOX + NIR group exhibited the most potent therapeutic efficacy, with tumor volume reduced to approximately 400 mm^3^ after 14 days, highlighting the strong synergistic effect between chemotherapy and photothermal therapy (PTT).

The superior performance of GHR/DOX is closely related to the unique physicochemical properties and biological behavior of GO nanomaterials *in vivo*. Graphene oxide possesses abundant oxygen-containing functional groups (carboxyl, hydroxyl, and epoxy groups), enabling efficient drug loading through π-π stacking, hydrogen bonding, and electrostatic interactions [35]. These interactions provide pH-responsive drug release, as acidic conditions disrupt hydrogen bonding and weaken π-π stacking, thus accelerating DOX release at the tumor site. Additionally, GO's conjugated sp^2^ carbon domains endow it with strong near-infrared (NIR) absorption and efficient photothermal conversion, enabling localized heat generation upon laser irradiation, which further accelerates drug release and enhances cancer cell apoptosis [36].

Beyond drug delivery and photothermal properties, GO also exhibits biodegradability and dynamic interactions within the biological microenvironment. Previous studies have shown that peroxidase-catalyzed oxidation and glutathione-mediated reduction play crucial roles in the biodegradation of GO under physiological conditions, which enhances its biocompatibility and clearance [37]. This controlled degradation not only improves the safety profile but also facilitates gradual release of therapeutic payloads over time.

To assess the toxicity of the material in mice, changes in mouse weight were monitored. The toxicity of the material in mice can be evaluated by the change of mouse weight. As can be seen from Fig. 5D, the weight of the mice in each group remained constant during the treatment, indicating that the material did not did not induce any adverse effects in the mice.

To investigate the anti-tumor mechanism of GHR/DOX, tumor tissues were collected, fixed and sectioned after 14 days of treatment, H&E and TUNEL staining were performed. As shown in Fig. 5E–H & E staining revealed that the GHR/DOX + NIR treated group caused the largest area of tumor tissue necrosis, most of the nuclei deformed, and the intercellular space enlarged. The results of TUNEL staining showed that different materials had different degrees of apoptosis to the DNA of tumor cells, and the injection of GHR/DOX + NIR had the most significant DNA damage, it also confirmed the best effect of tumor inhibition. Additionally, we employed CD31 and Ki67 of tumor sections to explore the effect on tumor vessel density and tumor tissue proliferation activity, respectively. CD31, also known as platelet-endothelial cell adhesion molecule with a molecular weight of 130 kDa, is primarily employed in immunohistochemistry to visualize endothelial tissue and assess tumor angiogenesis. [38]. The results revealed a significant reduction in tumor tissue vessel density following treatment with GHR/DOX + NIR, indicating effective inhibition of angiogenesis in tumor tissue. Ki67, a nuclear protein associated with ribosomal RNA transcription and serves as a marker of cell proliferation [39]. The Ki67 staining image shows that GHR/DOX + NIR significantly inhibited tumor cell proliferation activity.

Overall, these results demonstrate that the therapeutic performance of GHR/DOX benefits from the synergistic effect of targeted drug delivery, photothermal therapy, and responsive drug release, all of which are closely linked to the unique structural and chemical properties of GO. Combined with its biodegradability and favorable safety profile, this makes GHR/DOX a promising platform for safe and effective breast cancer therapy.

### 3.8 *In vivo* photothermal imaging and fluorescence imaging

**Fig. 6:**
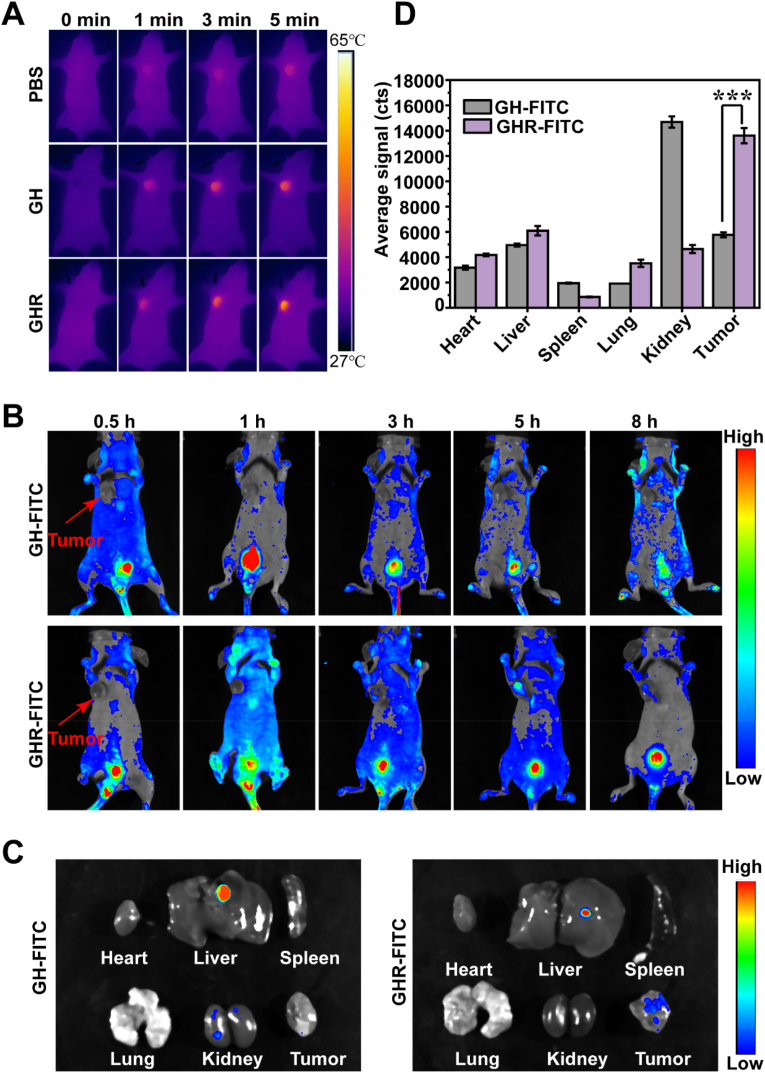
**(A)** Infrared thermographic maps of tumor site after irradiated with NIR light; (B) In vivo fluorescent imaging of tumor-bearing nude mice after intravenous injection of GH complexes with and without targeting RGD at different times; (C) fluorescent images of the main organs collected after 8 h treatment of GH and GHR; (D) Quantitative detection of fluorescent.

The photothermal effect *in vivo* is showed in Fig. 6A. The temperature of the PBS group barely changed with body temperature, while the temperature of the GH group began to rise gradually with time. Moreover, the temperature of GHR increased more significantly. These results indicate that GHR exhibits a good photothermal effect and targeting capability. The targeting ability of GHR was assessed by fluorescence imaging experiments, and the results are shown in Fig. 6B, compared with GH-FITC; GHR-FITC showed obvious fluorescence signal at the tumor site at 0.5 h after intravenous injection. There was no enrichment of fluorescence signal at the tumor site of nude mice injected with GH-FITC over time. However, due to the tumor-targeting effect of GHR-FITC, its fluorescence signal at the tumor site was significantly enhanced 3 h after injection. t These experimental results indicate that modification of RGD enhanced the tumor targeting of the material. At 8 h after injection, nude mice were sacrificed, and tumor and normal tissues were collected for ex vivo imaging. As can be seen from Fig. 6C and D, among the organs of the nude mice injected with the GH-FITC group, only the kidneys exhibited a fluorescence signal, while the tumor showed no fluorescence signal. However, the tumor site of the nude mice injected with GHR-FITC still exhibited a relatively strong fluorescence signal, further indicating that the modification with RGD increased the targeting of the material.

### 3.9 *In vivo* safety evaluation

**Fig. 7:**
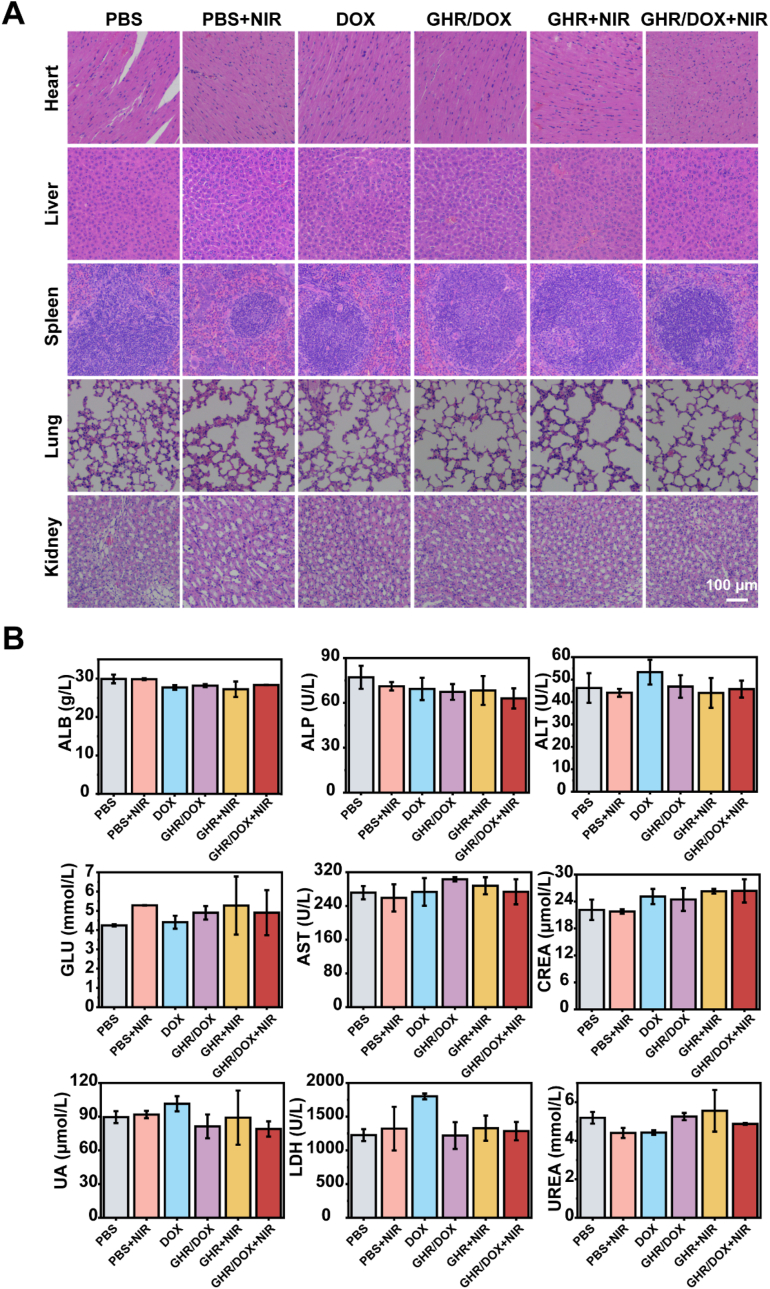
(A) Histologic assessments of major organs in mice treated with different formulations; (B) blood parameters for evaluating functions of the Albumin (ALB), lactate dehydrogenase (LDH), urea nitrogen (BUN), creatinine (CRE), total cholesterol, T-cho, serum total protein (TP), low-density lipoprotein (LDL), globulin (GLB) and glucose (GLU).

Biocompatibility is a prerequisite for the safe use of materials in nanomedicine. Therefore, we investigated the effects of different treatment groups on major organs and blood parameters in mice. As shown in Fig. 7A–H&E images of major organs from nude mice after 14 days of treatment of tumor-bearing nude mice in various experimental groups show no damage to tissue morphology or organ structure compared with the PBS group. This indicates that the materials used in the experiment did not induce any physiological toxicity in mice. Additionally, we measured and analyzed the routine blood parameters of nude mice in different treatment groups. As shown in Fig. 7B, no significant changes were observed in the blood indices of nude mice in the PBS, GHR/DOX, GHR + NIR and GHR/DOX + NIR treated groups compared with healthy mice. However, DOX caused a significant decrease in albumin (Alb) and low-density lipoprotein (LDH), indicating potential effects on kidney, liver, and myocardial function. In summary, GHR complexes exhibit good biocompatibility and can be safely used in tumor treatment.

## 4 Conclusions

In this study, we synthesized a photothermal nanomaterial via Michael addition and RGD peptide modification for loading chemotherapy drug DOX. Which have good photothermal effect and the ability to control drug release through NIR irradiation. *In vitro*, GHR/DOX demonstrated effective anti-tumor activity and promoted tumor cell apoptosis through the combined effects of photothermal and chemotherapy drugs. Additionally, which were specifically uptake by MCF-7 cells through the RGD peptide mediated cell internalization. *In vivo*, GHR/DOX exhibited excellent anti-tumor effects and specific targeting through photothermal and chemotherapy effects as well as RGD targeting. Therefore, the novel GHR/DOX can provide a new guiding significance for clinical treatment of breast cancer.

## CRediT authorship contribution statement

**Xiongjie Zhu:** Writing – original draft, Software, Project administration, Investigation, Funding acquisition, Data curation, Conceptualization. **Juanjuan Lei:** Writing – original draft, Validation, Software, Project administration, Methodology. **Chao Jiang:** Visualization, Resources, Project administration, Investigation, Formal analysis. **Zhaobi Fang:** Writing – original draft, Validation, Resources, Methodology. **Wenkai Zhang:** Visualization, Validation, Methodology, Investigation. **Zhe Yang:** Visualization, Validation, Supervision. **Rui Guo:** Writing – review & editing, Funding acquisition, Formal analysis, Conceptualization. **Rui Xu:** Writing – review & editing, Visualization, Funding acquisition, Data curation, Conceptualization. **Xiaoshan Hu:** Visualization, Supervision, Project administration, Funding acquisition, Conceptualization.

## Ethics approval and consent to participate

All animal experiments approved by the Institutional Animal Care and Use Committee of the Guangzhou medical University.

## Funding

This work was supported by the Scientific Project Foundation of Guangzhou City (No. 2023A04J1200), Basic Research Project of Science and Technology Program of Bao'an District, Shenzhen (Grant No. 2021JD042), and the Plan on Enhancing Scientific Research in GMU.

## Declaration of competing interest

The authors declare that they have no competing interests.

## Data Availability

Data will be made available on request.
