# Supplementary material for: Two-dimensional graphene nanomaterials for combined photothermal and chemotherapy-enhanced targeted therapy of breast cancer
Source: Mater Today Bio. 2025 Mar 13;32:101668. doi: 10.1016/j.mtbio.2025.101668 (PMC11957798; doi:10.1016/j.mtbio.2025.101668)
Supplement: Multimedia component 1 [file mmc1.docx]

**Supporting Information**

**Two-dimensional graphene nanomaterials for combined photothermal and chemotherapy-enhanced targeted therapy of breast cancer**

Xiongjie Zhu^1#^, Juanjuan Lei^2#^, Chao Jiang^3#^, Zhaobi Fang^4^, Wenkai Zhang^1^, Zhe Yang^5^, Rui Guo^6*^, Rui Xu^1*^, Xiaoshan Hu^1*^

^1^ Department of Medical Oncology, Guangzhou institute of Cancer Research, the Affiliated Cancer Hospital, Guangzhou Medical University, Guangzhou, China

^2^ Department of Hepatobiliary Surgery, Guangzhou institute of Cancer Research, the Affiliated Cancer Hospital, Guangzhou Medical University, Guangzhou, China

^3^ Department of Cancer Center, The People’s Hospital of Baoan Shenzhen, Guangdong, China.

^4^ Department of Oncology, Zhujiang Hospital of Southern Medical University, Guangzhou, China

^5^ Southern Medical University, Guangzhou, China

^6^ Key Laboratory of Biomaterials of Guangdong Higher Education Institutes, Guangdong Provincial Engineering and Technological Research Centre for Drug Carrier Development, Department of Biomedical Engineering, Jinan University, Guangzhou.

^#^ These authors contributed equally to the present research

^*^ Corresponding Authors: Xiongjie Zhu, E-mail: [doctorzhu2021@gzhmu.edu.cn](mailto:doctorzhu2021@gzhmu.edu.cn); Rui Xu, E-mail: gzxr@gzhmu.edu.cn; Rui Guo, E-mail: [guorui@jnu.edu.cn](mailto:guorui@jnu.edu.cn).


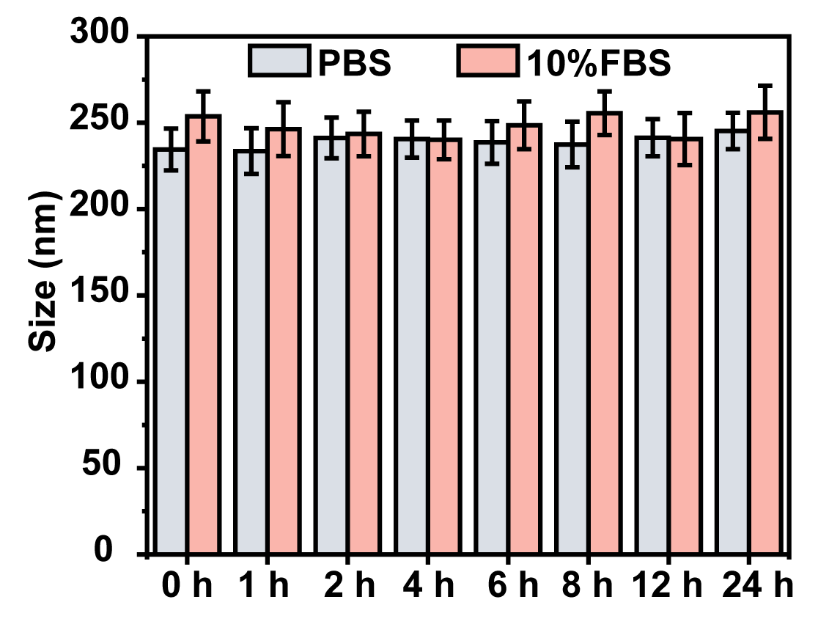


**Figure S1** The hydrated particle size of GO-HPAA changes with time under PBS or 10% FBS.


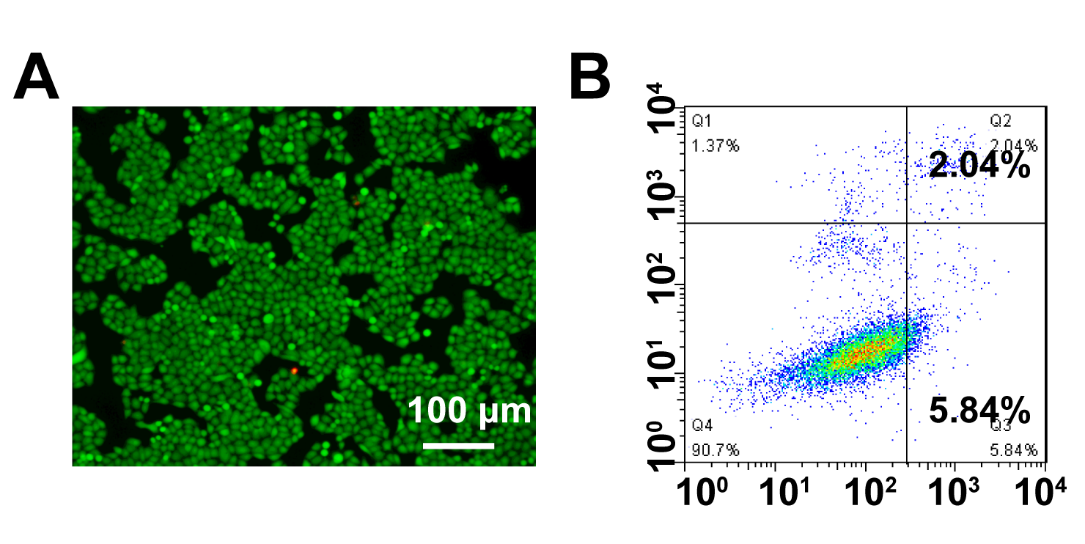


Figure S2 (A) The live-death staining image of the illumination group alone; (B) The flow cytometry image of cell apoptosis in the illumination group alone.
